# Can patient-physician interview skills be implemented with peer simulated patients?

**DOI:** 10.1080/10872981.2022.2045670

**Published:** 2022-03-01

**Authors:** Funda İfakat Tengiz, Hale Sezer, Aysel Başer, Hatice Şahin

**Affiliations:** a School of Medicine, Medical Education Department, Izmir Katip Çelebi Üniversitesi Tıp Fakültesi Tıp Eğitimi Anabilim Dalı; Izmir Katip Celebi University, Izmir, Turkey; b Faculty of Health Sciences, Nursing Department, Izmir Bakırçay Üniversitesi Sağlık Bilimleri Fakültesi Hemşirelik Bölümü; Izmir Bakırçay University, Izmir, Turkey; c School of Medicine, Medical Education Department, Izmir Demokrasi Üniversitesi Tıp Fakültesi Tıp Eğitimi Anabilim Dalı; Izmir Demokrasi University, Izmir, Turkey; d Medical Education Department, Ege Üniversitesi Tıp Fakültesi Tıp Eğitimi Anabilim Dalı, Ege University School of Medicine, Izmir, Turkey

**Keywords:** Patient-physician interview skills, peer-assisted learning, simulation, peer simulated patient, peer simulation

## Abstract

**Introduction:**

Patient-physician interviewing skills are crucial in health service delivery. It is necessary for effective care and treatment that the physician initiates the interview with the patient, takes anamnesis, collects the required information, and ends the consultation. Different methods are used to improve patient-physician interview skills before encountering actual patients. In the absence of simulated patients, peer simulation is an alternative method for carrying out the training. This study aims to show whether patient-physician interview skills training can be implemented using peer simulation in the absence of the simulated patient.

**Methods:**

This is a descriptive quantitative study. This research was conducted in six stages: identification of the research problem and determination of the research question, development of data collection tools, planning, acting, evaluation, and monitoring. The data were collected via the patient-physician interview videos of the students. The research team performed descriptive analysis on quantitative data and thematic analysis on qualitative data.

**Results:**

Fifty students participated in the study. When performing peer-assisted simulation applications in the absence of simulated patients, the success rate in patient-physician interviews and peer-simulated patient roles was over 88%. Although the students were less satisfied with playing the peer-simulated patient role, the satisfaction towards the application was between 77.33% and 98%.

**Discussion and Conclusion:**

In patient-physician interviews, the peer-simulated patient method is an effective learning approach. There may be difficulties finding suitable simulated patients, training them, budgeting to cover the costs, planning, organizing the interviews, and solving potential issues during interviews. Our study offers an affordable solution for students to earn patient-physician interview skills in faculties facing difficulties with providing simulated patients for training.

## Introduction

Medical students need to practice patient-physician interviews to develop essential clinical communication and clinical reasoning skills and find the necessary space to apply their basic professional skills [1]. Patient-physician interviewing skills have an important place in health service delivery. A good interview is crucial for effective diagnosis and treatment. Medical educators agree that medical students should be humane and have the necessary communication skills for patient-physician interview skills. However, for years, there has been uncertainty about the ways to achieve this learning goal [2]. Having students experience a mock patient-physician interview is considered the easiest method to accomplish this goal [2]. Methods based on small group activities, such as problem-based learning, role-playing, and simulated/standardized patient simulation, are used to improve patient-physician interview skills [2,3]. Today, it is a common and accepted method to conduct patient-physician interviews with simulated/standardized patients [1^,4–6^]. Simulated patients can be theatre actors, professional actors, trained volunteers (retirees, students, employees, etc.). There is no evidence that the simulated patient has to be a professional actor for the interview to be efficient [4,7]. There are certain advantages and disadvantages to interviewing simulated patients. Simulated patients offer a student-centered educational opportunity that is the closest to reality without time constraints. They can impersonate different patient profiles and conditions, allowing students to experience patients and cases that are difficult to encounter in real life [4,5]. On the other hand, using simulated patients also has disadvantages related to the cost or training requirements [8]. There may be difficulties finding proper simulated patients, training them, budgeting to cover the costs, planning, organizing the interviews, and solving possible issues during interviews [4,5,7–12]. Furthermore, the need to train faculty members` for simulated patient training, the time spent on it, corporate commitments, and, most importantly, the truth that it is not a sustainable method are some other downsides [4,5].

In modern medical education, to improve patient-physician interviewing skills, it has become imperative to use modernized, affordable and sustainable models, instead of teacher-centered and expensive methods with a traditional approach. Peer-assisted learning (PAL) serves this purpose [3,13,14]. One can define PAL as knowledge and skills acquisition through active help and support among peers. Peer trainers (tutors) are non-professional teachers who, by helping their friends, help themselves as well to have a broader understanding of the topic at hand [3,14,15]. Peer-assisted learning (PAL) has long been used informally in medical education by medical educators as an auxiliary tool for learning since its inclusion among the effective models in the literature [3,13,16]. The primary advantage of PAL is economizing resources. Another advantage is that it immensely reduces the burden of the faculty member. It increases the cultivation of a lifelong learning mentality for students, leads to continuous professional development, and enhances interest in an academic career, boosting skills such as leadership, coaching, confidence, and inner motivation [13,14,16,17]. Peer simulation is presented as a new concept that increases the advantages of PAL [5]. Peer simulation is a structured form of role-playing in which students train to play the patient role for their peers [5]. Having peer support in peer simulation (peer simulated patient) presents many advantages offered by PAL, and it has a positive effect on learning outcomes. Students learn together and from each other through peer simulation. Peer simulation is an alternative method to using simulated patients in preclinical applications. Playing the patient role in peer simulation is an opportunity to facilitate the development of empathy and culture-sensitive medical practice skills [5]. There are very few examples of professional skills training using peer simulation [5].

According to the literature, there are no examples in Turkey yet. In the medical school, where the study was carried out, patient-physician interview skills training was implemented in the second year. The patient-physician interview skills training goal was to teach students the proper way to start the interview, take and expand the anamnesis, inform the patient, and end the interview. There are no simulated/standardized patients in this medical school. For students to gain skills, a different teaching strategy, which is low cost but meets the same function, is required.

**Figure 1.**
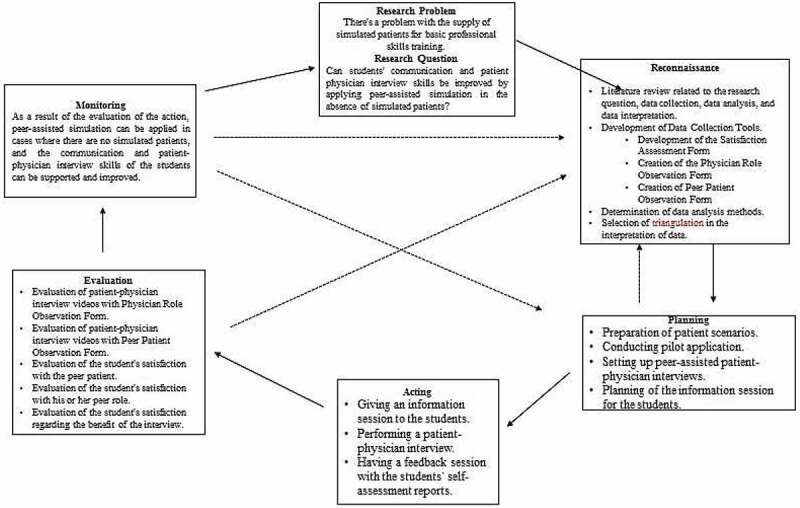
Mixed-Method Methodological Framework for Research.

In our school, action was planned to solve this problem. Results from action are the solution to the problem. Action research, used to improve and modify educational practices, is a method that helps faculty and students better understand the work carried out in the institution. If the results are not satisfactory, researchers retry [18]. The action process is carried out in six stages (Figure 1). The first stage is ‘diagnosing,’ which means identification of the problem. The second stage is ‘reconnaissance,’ in which data collection tools are developed and the problem is analyzed and interpreted. The third stage contains the development of the action/intervention plan. The acting stage includes the implementation of the action/intervention plan. The fifth stage is the evaluation stage comprising data collection and analyzing the action/intervention. The last stage includes monitoring the data to make revisions and test the action/intervention.

This study aims to show whether peer simulated patient-physician interview skills training can be successfully implemented to practice patient-physician interviewing skills of medical students in the absence of simulated patients.

## Methods

This is a descriptive quantitative study. With the descriptive methodological framework, the problem was subjected to a comprehensive initial assessment, and multiple data are collected and integrated. Thus, a more rigorous evaluation of the action was obtained [18–20]. In this study, first, the problem was defined, then data collection tools were developed with the support of literature, remedial action was planned, and finally, the developed training model was applied. The process of this research was carried out in stages and is shown in the figure (Figure 1).

Figure 1- Descriptive Methodological Framework for Research

The method of the research will be presented in accordance with the stages:

In the literature review ‘patient-physician interview skills, peer-assisted learning, simulation, peer-simulated patient, peer simulation’ keywords were used. Applications on peer-assisted learning and peer simulation were examined in 51 studies.

Based on the literature information, data collection tools aimed at obtaining the opinions of different parties have been developed to evaluate peer-assisted patient-physician interview skills.

i. Physician’s Role Observation Form (PROF).

Using the literature, the researchers identified observational headings related to patient-physician interview skills [4, 7, 21], Katharina Eva [22], Katharina Eva [1, 23–26]. After four consecutive meetings, the researchers reached a consensus on the identified headings. An observation form on patient-physician interview skills was created by grouping the agreed items in line with their conceptual similarities.

PROF consists of three groups (verbal communication, nonverbal communication, questioning of the main complaint) and 54 items. Each answer is rated as “0-no” for missing the objective and “1-yes” for reaching the objective.

ii. Peer Patient Observation Form (PPOF). Using the literature, the researchers identified headings related to the role of simulated patients [4,21,26]. The researchers agreed on PPOF consisting of eight items. Each answer is rated as “0-no”, “1-yes”.

iii. Satisfaction Assessment Form (SAF). The form consists of socio-demographic variables (four items), and items related to the satisfaction with the patient-physician interview (six items), and related to the peer-assisted patient-physician interview (15 items related to the physician’s role, three items related to the peer-simulated patient’s role, and three items related to the observer). All questions except two are closed-ended. Data on whether the peer-assisted patient-physician interview was beneficial was obtained by evaluating the open-ended questions of the SAF.

### Data analysis methods

Student interview videos were viewed separately by researchers. Each student received grades for their roles as a physician and a patient. Accordingly, a student playing the physician’s role received a minimum score of 0 and a maximum score of 54 from the *PROF*. The student playing the peer-simulated patient’s role received a minimum score of 0 and a maximum score of 8 from the *PPOF*. The internal consistency of the scales was evaluated with the Crohnbach’s alpha coefficient. For the analysis of the results from the *SAF*, descriptive analysis was performed for the answers to two open-ended questions, and frequency values and means were calculated in closed-ended questions. The statistical software SPSS 24 (Statistical Package for Social Sciences for Windows 24.0) was used for calculations.

In addition, it is aimed that students can reach all the gains in the expressions specified in the form. Therefore, the success-satisfaction ratio of the items on the form was calculated using the formula “number of successful-satisfied answered items/total number of items*100”. This ratio was calculated for the physician’s role observation form (54 items), peer simulated patient observation form (8 items), and the peer-assisted patient-physician interview satisfaction section (21 items) of the SAF.

### Planning

a. Preparation of simulated patient scenarios.

A patient scenario for history taking was created by the researchers using the literature. Scenario creation stages are as follows: the determination of learning objectives and outcomes, determination of context and content (the physician’s and patient’s roles, anamnesis information, physical environment, available source, etc.), evaluation of technical infrastructure (computer, camera, sound system), and preparation of supporting documents [27,28]. The scenario was submitted to the expert opinion and was made ready for application after making the necessary revisions. Patient scenarios, which were finalized with the feedback from expert, were prepared for information sessions with students.

b. Conducting pilot application.

The pilot application was conducted with eight volunteering second-year students who had no experience with interviewing simulated patients. Information sessions were held with the volunteering students, and patient-physician interviews were planned. Within the scope of the pilot application, volunteering students made interviews with their peers playing the physician’s role, patient’s role, interviews were video-recorded, and feedback sessions were held with students. Video recordings were evaluated by the researchers using data collection forms. Technical problems encountered in the pilot application (internet, computer screen resolution, sound quality, etc.) and data collection tools were fixed.

c. Setting up the peer-assisted patient-physician interview.

During the 2019–2020 academic year, second-year students, at Izmir Katip Çelebi University Faculty of Medicine participated in the peer-assisted patient-physician interviews. Throughout the module, a student had three different responsibilities: playing the physician’s role, playing the peer-simulated patient’s role, and being the peer observer. Thus, students were able to experience all the components of the interview directly. Students made interviews, which were video-recorded. After the interview, they filled out a satisfaction form, wrote a self-assessment report, and attended a feedback session. Those playing the patient’s role simulated the disease required by the role, monitored the interviewing physician, gave constructive feedback to the physician, and filled out the satisfaction form. Finally, those who acted as an observer monitored the physician’s performance, gave constructive feedback, and filled out the satisfaction form.

d. Planning a feedback session with students after the interviews.

Students watched a video recording of the interview, wrote the self-assessment report, and participated in the feedback session

### Acting

At this stage, patient-physician interviews were made, and information sessions were delivered about student responsibilities, and feedback sessions were held. Before this, second-year students who participated in basic communication skills, clinical communication skills, and professional skills courses had a patient-physician interview at the student outpatient clinic during appointment hours. The interviews were conducted simultaneously in five outpatient clinics by teams of five people. In these teams, one of the students played the physician’s role, one played peer-simulated patient’s role, and three participated in interviews as observers. In subsequent interviews, the students exchanged their roles: each student was allowed to play the physician’s and peer simulated patient roles once, and the observer roles three times. The student playing the physician’s role was required to prepare the outpatient clinic, initiate video recording, meet the patient, take anamnesis, and make general situation assessment. The student playing the peer-simulated patient’s role was informed that they could improvise if the answer to the question was not specified in the scenario. Observing students were required to monitor the interview and give feedback to the interviewing physician at the end. Once the interview was over, the student playing the physician’s role took the video recording, wrote the self-evaluation report, and participated in the feedback session held the following week. In the feedback session, the patient-physician interview experience was evaluated using discussion, reflection, and feedback techniques. This stage was completed in March 2020.

Student interview videos were monitored and analyzed by researchers with PROF, PPOF, and SAF.

The findings obtained after the analysis of the data were interpreted with triangulation, and a decision was made regarding the continuation of the peer-assisted simulated patient-physician interview. All data obtained by triangulation are combined and interpreted in a table.

### Ethics committee

Approval was obtained from the research ethics committee of the ICU Social Research Ethics Committee in March 2020 with the decision numbered 2020/03–04.

## Results

It was aimed to ensure that all second-year students (n:193) participated in patient-physician interviews. Patient-physician interviews were planned to be held throughout eight weeks according to a schedule, in which each week 25 students participated in the interviews. After the first two weeks, the COVID-19 pandemic was declared by the WHO, so the remaining students were unable to make the interviews. Thus, the interview videos of a total of 50 students were monitored by researchers and analyzed by obtaining data with PROF and PPOF. Cronbach’s alpha of PROF was found to be 0.71.

**Chart 1.**
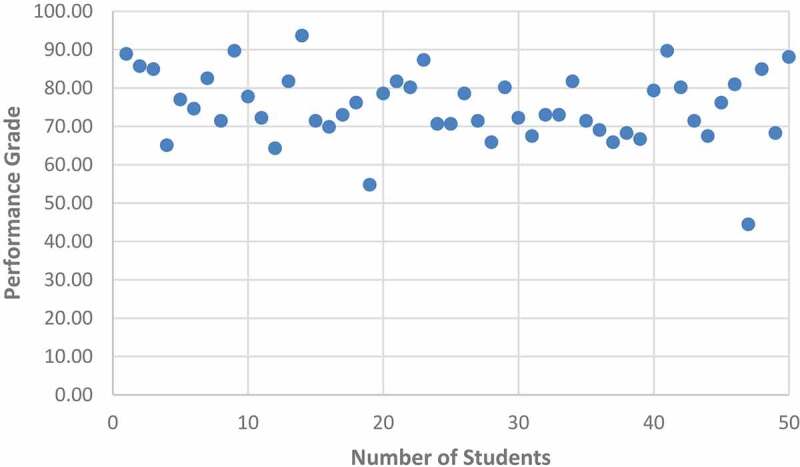
Students` performance grade distributions from PROF.

A total of 50 students (31 males and 19 females) participated in the study. The mean age of the students is 20.56 (min:19 max:23).

a. In the analysis of the data obtained from the patient-physician interview video recordings (n:50), the total score and success percentage for each student were calculated with PROF. Accordingly, the mean and standard deviation of the scores form PROF were 70.43 ± 9.81 (min. 40.12, maximum 88.27), respectively. Students are expected to get at least 60 points in order to be considered successful. The rate of students who were successful with a score of 60 or above from PROF was 92%. The distribution of achievement scores is presented as a graph (Chart 1).

Chart 1. Students` performance scores from the PROF

When evaluating the students playing the physician’s role, the headings on the PROF were examined: 96.29% of the 54 items were found to be used effectively during the observation. Students were successful in over 95% of the topics of welcoming patient, asking questions about the patient’s demographic characteristics, making eye contact, listening to the patient’s main complaints, observing the patient’s profile, and asking questions about background. On the other hand, students achieved less than 50% success in summarizing the case, using body language, using the proper tone of voice, and using understandable language.

b. Students playing the peer-simulated patient’s role were evaluated via the PPOF by considering patient-physician interview video recordings (n:50). Students were found to be more than 90% successful in seven items of the form. However, only 32% success was achieved in the eighth item related to the peer patient giving feedback to the interviewing physician, (Table 1).

**Table 1.** Peer-Simulated Patient Success Rate

PPOF Items	%
1. The peer patient focused on the script. (good recall, concentrated)	91,33
2. The peer played the role of patient well.	94,67
3. The peer patient was able to present alternative topics to the topics highlighted in the scenario	95,33
4. The oral communication skills of the peer patient were appropriate (clear, clear, understandable, scripted)	99,33
5. The nonverbal communication skills of the peer patient were appropriate (body language, gesture, gesture).	99,33
6. The peer patient listened to the physician interview topics effectively	100,00
7. The peer patient answered the questions of the interviewer consistently. (credible-reliable)	99,33
8. The peer patient gave effective feedback.	32,00
Total	88,92

c. Findings regarding the satisfaction with the peer-assisted patient-physician interview were presented under the following headings: sociodemographic characteristics of the participants, their opinions on satisfaction with the patient-physician interview, and their opinions on satisfaction with the peer-assisted patient-physician interview.

After the evaluation on the satisfaction of the peer-assisted patient-physician interview, it was determined that 98% (n:49) of the students were satisfied with the peer-assisted patient-physician interview, and 84% (n:42) were satisfied with the presence of their peers in the patient role in the peer-assisted patient-physician interview. The other, 16% (n:8) stated that they would prefer to have an real patient or doctor instead of their peers. It was also determined that 92% of the students wanted to re-experience the peer-assisted patient-physician interview in the coming years, and 96% found the peer-assisted patient-physician interview experiences useful.

Regarding their answers to the open-ended questions, the students stated that they found it valuable to have experienced the patient-physician interview in the early period during the pre-graduation medical education process. They noted that they realized their weaknesses and what needed to be done about them. They said that it would be useful to repeat this instructive practice, that peer-assisted learning was valuable, and that it was a good opportunity to self-evaluate. On the other hand, some of the negative remarks related to the process were inexperience, excitement, personal inadequacies, lack of knowledge, unnecessary role-playing, and difficulty communicating with the patient”.

When the satisfaction with peer-assisted patient-physician interviews was evaluated, it was determined that 77.33% of the students were satisfied. 80.53% of the students were satisfied with being interviewing physician, 56.66% with being the peer-simulated patient, and 82% with being observer in the interviews.

d. All the data obtained is combined with triangulation and combined and interpreted in the table.

In triangulation, the students playing the physician’s, and patient’s roles were evaluated together with ‘success in being simulated patients’ and ‘satisfaction with the peer-assisted patient-physician interviews’(Table 2).

**Table 2.** Triangulation of Patient-Physician Interview Skills Data

Merged Data		%
Success Rate of Being an Interviewer Physician		92,00
Peer Simulated Patient Success Rate		88,92
PatientPhysicianInterviewSatisfactionRate	Satisfaction with patient-physician interview	98,00
	Satisfaction with the fact the simulated patient is a peer simulated patient	84,00
	Interest to have a patient-physician interview in the years to come	92,00
	Finding the patient-physician interview experience helpful	96,00
	Finding the patient-physician interview experience useful	77,33
	Satisfaction of being an interviewer physician Peer-to-peer simulated patient satisfaction Satisfaction of being an observer	80,53 56,66 82,00

In the absence of simulated patients, it was determined that students achieved an over 88% success rate in the patient-physician interviews and peer-simulated patient roles. Although they were less satisfied with playing the peer-simulated patient’s role, the satisfaction with the peer-assisted patient-physician interviews was rated between 77.33% and 98%.

## Discussion

This study was conducted to determine whether medical students’ patient-physician interview skills could be implemented by peer simulation in the absence of simulated patients.

In faculties facing difficulties with providing simulated patient for patient-physician interview skills training, a different teaching strategy that meets the same function is needed to ensure that students gain skills at a low cost. Indeed, in this study, nearly all of the students were successful in patient-physician interviews performed using peer-simulated patients.

The 26,found that changing a student’s role during learning experiences encourages students to learn [26]. In another study conducted with peers, it was determined that patient-physician interviews contributed to the students’ ability to take anamnesis, manage emotional problems, and self-assess [5, 23]. Similarly, peer simulation develops communication, empathy, trust, and professional skills [5]. In our study, we observed that students playing the physician’s role were successful in starting patient interviews, taking anamnesis, and using the appropriate nonverbal communication skills. These students were evaluated through the PROF, which Cronbach’s alpha reliability coefficient was found to be 0.71. In the literature, Cronbach’s alpha reliability coefficient is interpreted as good if it is between 0.70 and 0.90 [29].

1,and 30,emphasized that design features such as feedback, planned implementation, the difficulty of simulation, clinical variation, and individualized learning should be taken into account in simulation training [1,30]. In our study, it was seen that students playing the peer-simulated patient’s role failed to give feedback to those playing the physician’s role. However, although the students were trained in giving feedback, they were found to be biased. 31,emphasized that peers evaluated each other generously in peer evaluation, while another study stated that peers may rate each other highly in small groups (small circle collusion) or large groups (pervasive collusion) [31,32].

In studies related to patient-physician interviews performed with peer simulation method, it is said that students can carry out the training process more easily than they do with simulated patients as they play the peer-simulated patient’s role [5]. In our study, while playing the physician’s and observer’s roles was satisfactory for the students, playing the peer-simulated patient’s role was not that satisfactory. One can speculate that they had difficulty getting into the role, as the patient-physician interview skills training using the peer-simulation method was conducted for the first time. It is thought that students’ satisfaction may increase as they become more familiar with the patient-physician interview skills training.

During peer simulation, students contribute to each other’s learning ‘as patients’ not by ‘teaching’ [5]. 7,similarly state that students could develop the ability to conduct patient-physician interviews if they observed other physicians [7]. In our study, students expressed their satisfaction and contribution to their learning by playing the observer’s role.

According to the systematic review of the studies that perform patient-physician interviews with peer-simulated patients, peer simulation is an effective learning approach [5]. In our study, as a result of the evaluation of the action, the patient-physician interviews with the peer-simulated patient was successfully completed.

One limitation of this study is failing to practically compare the peer simulation technique with standardized patient simulation due to the lack of standardized patient simulation in the medical school where the application was carried out. Another limitation is the inability to include all second-year students in this study due to the pandemic.

## Conclusion

In the absence of simulated patients, peer-assisted simulation can be performed to contribute to medical students’ patient-physician interview skills. To obtain better results from peer-assisted patient-physician interviews, making the following arrangements within institutions is recommended:

• Organizing additional training to increase students’ ability to give constructive feedback to their peers,

• Planning multicenter researches that evaluate the institution gains (time, cost, workforce, etc.) obtained through peer-simulated patient usage.

• Ensuring the sustainability of the action research cycle by evaluating peer-simulated patient practice in the coming years.

Consideration of peer-assisted simulation by educators, students and administrators will ensure that the practice becomes widespread.
